# Plexin C1 Marks Liver Cancer Cells with Epithelial Phenotype and Is Overexpressed in Hepatocellular Carcinoma

**DOI:** 10.1155/2018/4040787

**Published:** 2018-09-19

**Authors:** Gorkem Odabas, Metin Cetin, Serdar Turhal, Huseyin Baloglu, A. Emre Sayan, Tamer Yagci

**Affiliations:** ^1^Department of Molecular Biology and Genetics, Gebze Technical University, Kocaeli, 41400, Turkey; ^2^Department of Medical Oncology, Anadolu Medical Center, Gebze-Kocaeli, 41400, Turkey; ^3^Department of Pathology, Anadolu Medical Center, Gebze-Kocaeli, 41400, Turkey; ^4^Faculty of Medicine, Cancer Sciences, University of Southampton, Southampton, SO16 6YD, UK

## Abstract

**Background and Aims:**

Hepatocellular carcinoma is an aggressive malignancy of the liver and is ranked as the sixth most common cancer worldwide. There is still room for novel markers to improve the diagnosis and monitoring of HCC. Our observations in cancer databases that* PLXNC1* is upregulated in HCC led us to investigate the expression profile of Plexin C1 mRNA and protein in HCC cell lines and tissues.

**Methods:**

A recombinant protein encompassing part of the extracellular domain of Plexin C1 was used as an antigen for monoclonal antibody development. Transcript and protein levels of Plexin C1 in HCC cell lines were determined by RT-qPCR and Western blotting, respectively.* In vivo* evaluation of Plexin C1 expression in HCC tissues was accomplished by immunohistochemistry studies in tissue microarrays.

**Results:**

A monoclonal antibody, clone PE4, specific to Plexin C1, was generated.* In silico* and* in vitro* analyses revealed a Plexin C1-based clustering of well-differentiated HCC cell lines. Staining of HCC and nontumoral liver tissues with PE4 showed a membrane-localized overexpression of Plexin C1 in tumors (p=0.0118). In addition, this expression was correlated with the histological grades of HCC cases.

**Conclusions:**

Plexin C1 distinguishes HCC cells of epithelial characteristics from those with the mesenchymal phenotype. Compared to the nontumoral liver, HCC tissues significantly overexpress Plexin C1. The newly generated PE4 antibody can be evaluated in larger HCC cohorts and might be exploited for the examination of Plexin C1 expression pattern in other epithelial malignancies.

## 1. Introduction

Hepatocellular carcinoma (HCC) is the fifth most common cancer among man and seventh among woman and ranked as the third most common cause of cancer-related deaths [1, 2]. Chronic liver injury, Hepatitis B (HBV) and C (HCV) virus infections, alcohol-associated diseases, and aflatoxin intoxications are the leading causes of HCC development. Several mutations affecting WNT/*β*-Catenin, PI3K/RAS, and oxidative stress signaling pathways as well as hot spot mutations in TP53 were frequently observed in hepatocarcinogenesis [3]. Orthotopic liver transplantation (OLT) is the most effective treatment option for patients with early diagnosis, albeit not suitable for advanced cases [4]. Despite the approval of two tyrosine kinase inhibitors, namely, sorafenib and regorafenib, for advanced cases, 5-year survival of HCC patients is only less than 20% [1].

Plexins comprise a large family of receptor proteins initially described in the nervous system [5]. Their ligands are semaphorins (Sema), a group of membrane-anchored or secreted proteins involved in several cellular functions including axon guidance, cell adhesion, and motility that altogether drive the development of tissues and organs [6]. Plexin receptors are characterized by their extracellular SEMA, PSI (Plexins, Semaphorins, and Integrins) and G-P (Glycine-Proline rich) domains and are divided into four subfamilies: Plexin A1-4, Plexin B1-3, Plexin C1, and Plexin D1 [5, 6]. Plexin C1, previously known as VESPR (Virus Encoded Semaphorin Protein Receptor), is a receptor for soluble viral semaphorin A39R originating from Vaccinia virus, which stimulates cytokine production from monocytes [7]. Later on, Tamagnone et al. reported that human ligand for Plexin C1 is the GPI-anchored Semaphorin 7A (Sema 7A) [5]. Increased phospho-cofilin level but decreased focal adhesion kinase (FAK) phosphorylation upon binding of A39R to Plexin C1 on mouse dendritic cells induced actin cytoskeleton rearrangement, which resulted in the inhibition of integrin-mediated adhesion as well as impaired chemokine-induced migration* in vitro* [8]. However, Sema 7A binding to Plexin C1 on melanocytes increased phosphorylation of both cofilin and FAK and total LIMK2 protein levels as well. These findings suggested that Plexin C1 may act as a tumor suppressor during melanoma progression through phosphorylation-mediated inactivation of cofilin [9]. Interestingly, Plexin C1 was found to impede Sema 7A functions that emerge from its binding to *β*1Integrin, another receptor for Sema 7A. Plexin C1 inhibited Sema 7A-*β*1Integrin mediated events including spreading and dentricity of melanocytes [10], and migration capacity of GN11 immortalized mouse cells secreting GnRH-1 [11]. Like other Plexins, Plexin C1 has a RAS-GAP activity evidenced by the fact that COS-7 cells transfected with cytoplasmic region of Plexin C1 display decreased R-RAS activity and their ECM-mediated migration decreases in a ligand-independent manner [12]. Sema 7A mediated axon outgrowth was shown to be mediated through *β*1-Integrin and independent of Plexin C1 [13], yet upregulated Plexin C1 transcript levels were found in especially early phases of neuronal development of rats [14]. Immunohistochemistry studies revealed that 66% of the metastatic melanoma tissues are devoid of Plexin C1 protein expression, while nontumoral adjacent tissues have modest to high level of Plexin C1 [9]. In line with this, Stirewalt et al. have found that Acute Myeloid Leukemia (AML) cells display decreased* PLXNC1* transcript levels when compared to normal hematopoietic cells [15]. Besides its prominent role in nervous system development, the aforementioned studies indicated differential expression of Plexin C1 in human malignancies. However, the expression of Plexin C1 in HCC cell lines and tissues and its role in hepatocarcinogenesis have not been defined so far. Therefore, we investigated Plexin C1 expression at both transcriptional and protein levels in HCC and studied its expression pattern in liver tissues by using a homemade anti-Plexin C1 monoclonal antibody.

## 2. Materials and Methods

### 2.1. Cell Culture and Reagents

HCC cell lines PLC/PRF/5, HEP3B, HEPG2, HUH7, and SK-HEP1 were maintained in low-glucose DMEM medium supplemented with 10% fetal bovine serum (FBS), nonessential amino acids, and antibiotics. SNU387, SNU398, and SNU423 cells were cultured in RPMI medium supplemented with 10% FBS and antibiotics. HEK293T cell line, SP2/0 mouse myeloma cells, and monoclonal anti-Plexin C1 antibody-secreting hybridoma cells were cultured in high glucose DMEM supplemented with 10% FBS and antibiotics. All cells were grown in a humidified incubator maintained at 37°C and 5% CO_2_ atmosphere.

### 2.2. In silico Analyses

Plexin C1 transcript levels in HCC were analyzed at Oncomine database (https://www.oncomine.org/resource/login.html) across “Chen Liver” microarray data filtered through “Hepatocellular Carcinoma vs. Normal” selection (104 HCCs vs. 76 liver tissues) [16]. In order to determine the expression of Plexin C1 transcript levels in epithelial vs. mesenchymal HCC cell lines, a search at “EMBL-EBI Expression Atlas” website (https://www.ebi.ac.uk/gxa/home) for* PLXNC1*,* CDH1*,* VIM*, and* PRKCA* genes on* Homo sapiens* dataset with “Cell Line” and “CCLE-Hepatocellular carcinoma” filters was accomplished. The output was downloaded and analyzed on R (3.3.3) to generate a heat map.

### 2.3. Production of shPLXNC1 Lentiviral Particles and Transduction PLC/PRF/5 Cells

Lentiviral particles were produced as follows: first, lentiviral* PLXNC1* shRNA (TRCN0000060645, Sigma-Aldrich, St. Louis, MO, USA) or control pLKO.1 (Addgene #8453) plasmids were mixed with packaging plasmids pCMV-dR8.2 dvrp (Addgene #8455) and pCMV-VSV-G (Addgene #8454) at a ratio of 1,5:1,5:1 in 250 *μ*l Optimem (Thermo Fisher Scientific, Rockford, IL, USA). Then, a second mixture consisting of the transfection agent PEI (Polysciences, Germany), which was added to 250 *μ*l Optimem at a ratio of 1:3 (DNA *μ*g: PEI *μ*l), was prepared. The two mixtures were assembled in a single tube to generate a transfection reagent, which was used to transfect HEK293T cells after incubation for 20 min at room temperature. After 36 hours, viral particles were harvested from the supernatant of the transfected cells, filtered through 0.45 *μ*m, and stored at -80°C. 1.5x10^5^ PLC/PRF/5 cells plated into a 6-well plate were transduced with viral particles in the presence of 8 *μ*g/ml Polybrene (Thermo Fisher Scientific). The next day, selection of transduced cells was initiated with the addition of 2 *μ*g/ml puromycin (InvivoGen, San Diego, CA, USA) into the culture medium.

### 2.4. Production of the Recombinant Protein

A partial recombinant protein encompassing the extracellular 66-274 aa. of the protein was produced as previously described [17]. Briefly, the coding region of* PLXNC1* corresponding to extracellular protein domain between 66 and 274 aa was cloned into pET101/D (Invitrogen, Carlsbad, Ca, USA) vector with an N-terminal 6-histidine tag. Recombinant protein was produced in Escherichia coli (BL21) and purified under denaturing conditions using Ni–NTA resin (QIAgen, Valencia, CA, USA). Refolding of the purified protein was performed by buffer exchange to phosphate buffered saline (PBS) by using NAP buffer exchange columns (Amersham, Piscataway, NJ, USA). Finally, the pure recombinant protein was concentrated using Centripreps centrifugal filters (Millipore, Billerica, MA, USA).

### 2.5. Monoclonal Antibody Production

8-10-week-old BALB/c mice were first immunized with 50 *μ*g recombinant protein emulsified in Complete Freund's Adjuvant (Sigma-Aldrich) and then the following injections were carried out every 3 weeks with recombinant protein mixed with Incomplete Freund's Adjuvant (Sigma-Aldrich). Sera of the immunized and control mice were tested for reactivity against recombinant Plexin C1 with indirect ELISA after third and fourth immunizations. Briefly, ELISA plates were coated with 100 ng of recombinant Plexin C1 protein in carbonate buffer (pH: 9,6). Mice sera were serially diluted and assessed for their immunoreactivity with Plexin C1 protein. Alkaline phosphatase-conjugated goat anti-mouse IgG (Sigma-Aldrich) was used as secondary antibody (1:1000). Colorimetric reaction developed upon addition of the substrate para-nitrophenyl-phosphate (Sigma-Aldrich) was measured at 405 nm in Varioscan Flash plate reader (Thermo Fisher Scientific). The mouse with the highest immunoreactivity against Plexin C1 was further boosted three days before the fusion. The fusion of freshly isolated splenocytes with SP2/0 myeloma cells was performed as described previously [18]. After fusion procedure, the cells were seeded in 96-well plates and then were selected first with HAT and then with HT. After single cell subcloning of the hybridoma cells, specific clones were expanded in culture and hybridomas were stored in liquid nitrogen. Antibody isotype was determined by Pierce Rapid Antibody Isotyping Kit (Thermo Fisher Scientific) according to manufacturer's instructions.

### 2.6. Purification of the Antibodies

Anti-Plexin C1 monoclonal antibodies were purified from hybridoma supernatants on AKTA-Purifier Chromatography (GE, Massachusetts, USA) using protein G affinity column (HiTrap protein G, GE). 50 ml hybridoma supernatant was loaded on the Protein G column, which was washed rigorously with sodium phosphate buffer (pH: 7,2). The bound antibodies were eluted with 0,1 M Glycine-HCI (pH: 2,7) and eluate fractions were neutralized with 50 *μ*l 1.5 M Tris-HCI (pH: 9,5). Purified antibodies were stored in aliquots at -20°C.

### 2.7. Western Blotting

Cell lysates were prepared by incubating cells on ice for 20 min in Triton X-100 lysis buffer [50 mM Tris–HCl pH: 8.0, 150 mM NaCl, 1% Triton X-100 with complete protease inhibitor cocktail tablets (Roche Diagnostics, Mannheim, Germany)]. Cells were scraped, transferred to microcentrifuge tubes, and incubated for 20 min on ice with occasional shaking. Then, cell debris was discarded by centrifugation at 18000 g and protein-containing supernatants were collected and stored at -80°C until use. The protein concentration of the samples was determined by a fluorometric assay using Qubit 3 Flourometer (Thermo Fisher Scientific). 50 *μ*g total protein samples were separated by SDS-PAGE gel and the transfer of proteins to PVDF membrane (Thermo Fisher Scientific) was performed with a semidry Trans-Blot Turbo Transfer System (Bio-Rad Laboratories, Hercules, Ca, USA). Membranes were first blocked with 5% skim milk in TBS containing 0.05% Tween-20 (TBS-T) and then were incubated overnight at 4°C with primary antibodies against Plexin C1 (1*μ*g/ml, clone PE4) and *α*-tubulin antibodies (Cell Signaling Technology, 1:10000) for equal loading control. After washing 3 times with TBS-T, membranes were treated with the HRP-conjugated goat-anti-mouse IgG secondary antibody (Cell Signaling Technology, Danvers, MA, USA, 1:10000) and washed again with TBS-T for 3 times. Protein bands were developed by using the Chemiluminescent Substrate Supersignal West Femto ECL (Thermo Fisher Scientific) and visualized using a ChemiDoc XRS system (Bio-Rad).

### 2.8. Immunofluorescence Assay

5x10^4^ cells were cultured on glass coverslips in 24-well plates overnight and fixed with 100% cold methanol on ice for 1 h. Fixed cells were blocked with 2% BSA in TBS for 45 min at room temperature. Cells were first incubated with the anti-Plexin C1 antibody (1:50) in blocking buffer containing 0.05% Tween-20 (BSA-T) for 1 h at room temperature and then with Alexa Fluor 488-conjugated anti-mouse IgG (Cell Signaling Technology) secondary antibody (1:500, in BSA-T) for 1 h at room temperature. After washing with PBS-Tween-20 the coverslips were mounted on glass slides with Prolong Gold antifade medium (Invitrogen) and sealed with nail polish. The staining was visualized using a Zeiss LSM 800 Airyscan Confocal Microscope (Zeiss, Germany).

### 2.9. RNA Isolation and RT-PCR

Total RNA was isolated from cell lines using a NucleoSpin RNA Plus kit (Macharey-Nagel, Düren, Germany). cDNAs were synthesized using a Protoscript M-MulV Taq RT-PCR kit (New England Biolabs, Massachusetts, USA) according to manufacturer's protocol. Real-time quantitative PCR (RT-qPCR) reactions were performed using a Maxima SYBR Green qPCR master mix (Thermo Fisher Scientific), including 0.2 *μ*M primers and 50 ng cDNA in a total volume of 20 *μ*l. The PCR reactions were started by an initial denaturation at 95°C for 10 min, followed by 45 cycles each consisting of 15 s denaturation at 95°C, 30 s annealing at 60°C, and 30 s extension at 72°C. Relative expression of* PLXNC1* mRNA in HCC cell lines was measured by normalizing* PLXCN1* expression to that of* GAPDH* and calculated with 2^−∆Ct^ formula [ΔCt = Ct (PLXNC1) - Ct (GAPDH)]. The primers for* PLXNC1* were 5′-AACTGTTCCCTTCCTTGACTAC-3′ and 5′-TCGTTGGCGTCTCTGTTATG-3′ and sequences of primers for* GAPDH* were 5′-GGCTGAGAACGGGAAGCTTGTCAT-3′ and 5′-CAGCCTTCTCCATGGTGGTGAAGA-3′.

### 2.10. Tissue Microarray (TMA) and Immunohistochemistry

In order to determine the protein levels of Plexin C1* in vivo*, HCC TMA slides containing 90-paired tumoral and adjacent normal tissues were purchased from US Biomax (Rockville, MD, USA). Tissue array slides were deparaffinized first at 70°C and then in xylene. After rehydration in graded alcohol series, glass slides were immersed in 10 mM citrate buffer, pH 6.0, and transferred into a microwave oven for 20 min for antigen retrieval. Endogenous peroxidase was blocked by incubation of slides in 3% H_2_O_2_ for 30 min [17]. Immunohistochemical staining of the tissues was carried out in an autostainer (BenchMark-XT, Ventana Medical Technologies, Roche Diagnostics). Briefly, tissues were first incubated with the anti-Plexin C1 monoclonal antibody (1:100) or with isotype antibody (Cell Signaling Technology) for 30 min at room temperature, and then with the secondary HRP-conjugated anti-mouse IgG antibody (Cell Signaling Technologies). The probed proteins were then visualized using chromogenic substrate 3,3′-diaminobenzidine (DAB) and examined under a Leica DM IL LED microscope (Leica Microsystems, NJ, USA). Photographs were taken with a microscope-attached Leica MC170 HD camera (Leica Microsystems). The level of Plexin C1 staining was determined by the histoscore (H-score) calculated by the multiplication of intensity score (0 = none, 1 = weak, 2 = moderate, and 3 = strong) with values representing the percentage of positively stained cells (0 = <10%; 1= 10-25%; 2= 25–50%; 3= 50 –75%; and 4= >75%). For chi-square analyses patients were grouped according to their H-score as follows: 0 = negative, 1-6 = weak, and 7-12 = strong.

### 2.11. Statistical Analyses

Paired Student's t-test was used for statistical analyses of immunohistochemistry scores. Chi-square analyses were performed to determine the correlation of Plexin C1 reactivity of HCC tumors with the clinicopathological characteristics of patients. Significant differences were denoted as follows: *∗*p < 0.05, *∗∗*p < 0.01, and *∗∗∗*p < 0.001.

## 3. Results

### 3.1. In Silico Analyses of PLXNC1 Transcripts on HCC Cell Lines and Tissues

**Figure 1:**
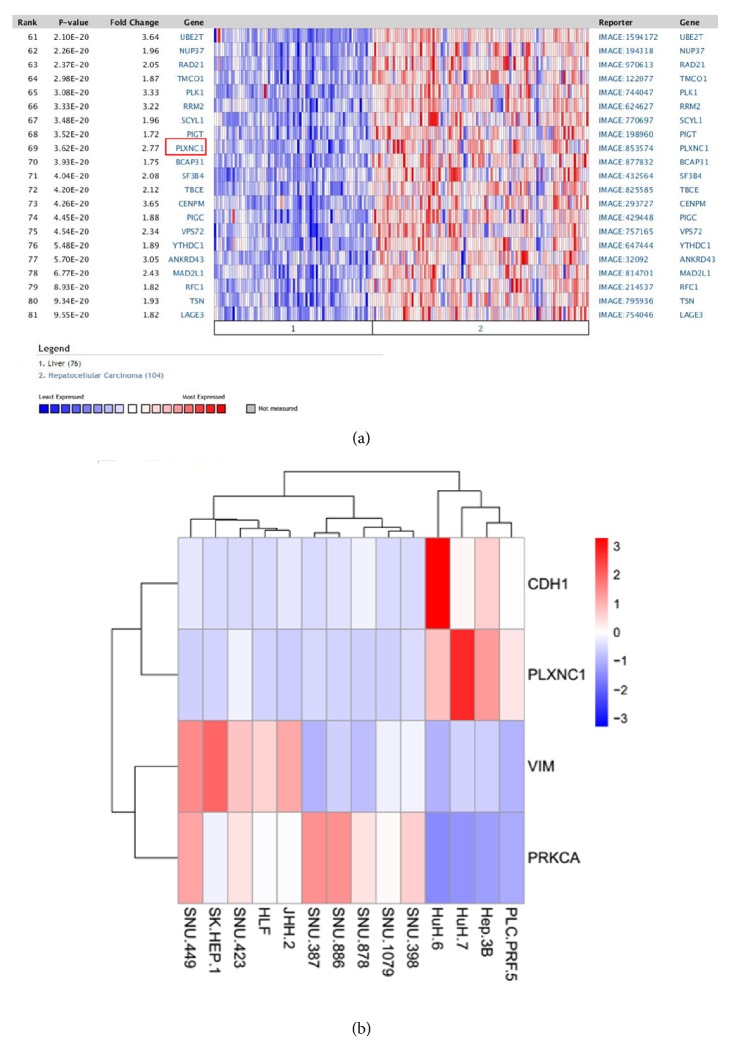
*In silico* analyses of* PLXNC1* expression. (a) Chen Liver microarray data compares the expression of 10802 genes between 104 HCC and 76 normal liver tissues. The mean of* PLXNC1* mRNA levels in HCCs is 2.77 fold higher than the mean of normal tissues. (b) Segregation of HCC* cell* lines with respect to their epithelial versus mesenchymal characteristics is analyzed on “EMBL-EBI Expression Atlas” database through the transcript levels of* PLXNC1*,* CDH1*,* PRKCA*, and* VIM* in HCC cells. HCC cells with epithelial phenotype include PLC/PRF/5, HEP3B, HUH7, and HUH6. HCC cell lines with mesenchymal phenotype are SNU398, SNU1079, SNU878, SNU886, SNU387, JHH2, HLF, SNU423, SK-HEP1, and SNU449.

In order to determine* PLXNC1* expression levels in HCC, “Chen Liver” microarray dataset (containing 10,802 measured genes through 76 normal and 104 HCC samples) deposited on Oncomine database was analyzed [16].* PLXNC1* mRNA expression was statistically higher (p=3.62e-20) in HCC tissues than in nontumoral adjacent tissues (Figure 1(a)). The differential expression of* PLXNC1 *between HCC and normal tissues prompted us to investigate whether* PLXNC1* transcripts segregate the differentiation status of HCC cell lines. To this end, we explored on “EMBL-EBI Expression Atlas” database the expression of* PLXNC1* in HCC cell lines along with the epithelial marker* CDH1* and the mesenchymal markers* PRKCA* and* VIM* genes. Interestingly,* PLXNC1* expression clustered HCC cells with epithelial characteristics with a distinction capacity comparable to* CDH1*. In sharp contrast, no* PLXNC1* expression was found in HCC cells displaying a mesenchymal phenotype (Figure 1(b)).

### 3.2. Differential Expression of Plexin C1 in HCC Cell Lines

**Figure 2:**
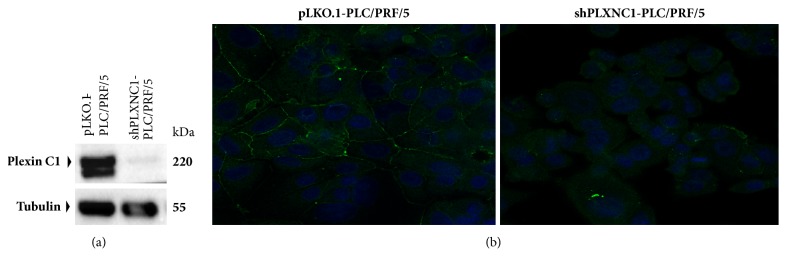
Validation of anti-Plexin C1 monoclonal antibody PE4. (a) Total proteins from PLC/PRF/5 cells transduced with shPLXNC1 and pLKO.1 empty control lentiviral particles are resolved on SDS-PAGE and Western blotting is carried out with the PE4 monoclonal antibody. Antitubulin monoclonal antibody is used for loading control. (b) Representative images from indirect immunofluorescence staining of pLKO.1-PLC/PRF/5 and shPLXNC1-PLC/PRF/5 cells with PE4 monoclonal antibody are shown.

**Figure 3:**
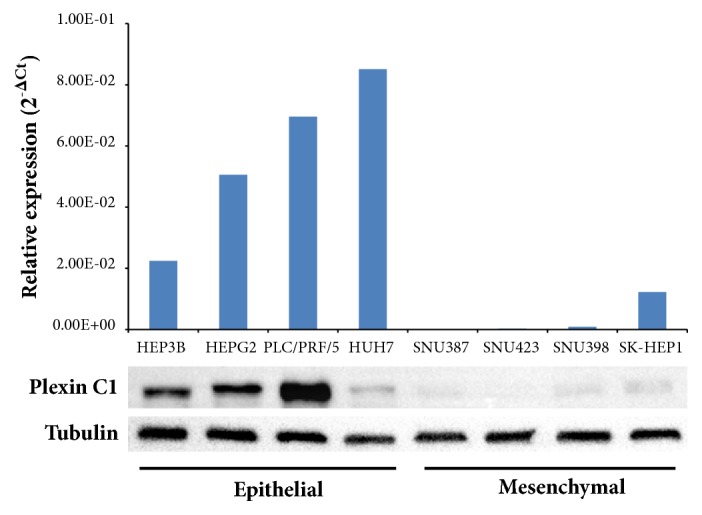
Expression of Plexin C1 transcripts and proteins in HCC cell lines. Epithelioid HEP3B, HEPG2, PLC/PRF/5, and HUH7 and fibroblast-like SNU387, SNU423, SNU398, and SK-HEP1 cells are analyzed. The upper diagram shows that the relative mRNA expression levels of* PLXNC1* and lower image display the protein expression profile of Plexin C1. Plexin C1 protein is detected using the PE4 monoclonal antibody. Tubulin is used for equal loading control.

To expand our analyses of Plexin C1 expression in HCC cells and tissues, we generated a monoclonal antibody of IgG2a isotype against a partial recombinant protein encompassing 66-274 aa. of Plexin C1 extracellular domain. Designated as PE4, we tested the specificity of this monoclonal antibody in shPLXNC1 knockdown PLC/PRF/5 HCC cells (Figure 2(a)). Further, in immunofluorescence microscopy, PE4 stained the membrane of pLKO.1-PLC/PRF/5 but the signal disappeared upon silencing* PLXNC1* expression by lentiviral shPLXNC1 transduction (Figure 2(b)). Next, to validate* in silico* data, the relative expressions of* PLXNC1* were measured in HCC cell lines by RT-qPCR and the protein levels of Plexin C1 were detected with PE4 antibody in Western blotting experiments. Consistent with our findings in* in silico* analyses, we observed in both assays higher expression of Plexin C1 in PLC/PRF/5, HEP3B, HEPG2 and HUH7 HCC cells with epithelioid phenotype compared to its highly downregulated expressions in SNU387, SNU423, SNU398 and SK-HEP1 HCC cell lines with mesenchymal characteristics (Figure 3).

### 3.3. Plexin C1 Is Significantly Overexpressed in HCC Tissues

**Figure 4:**
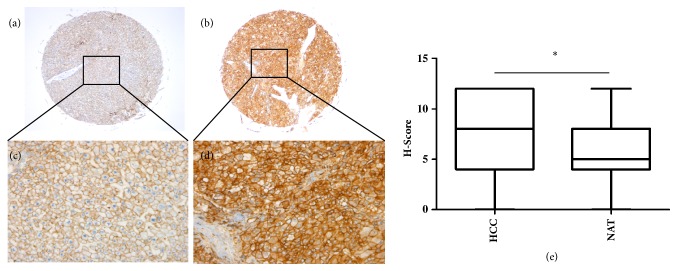
Immunohistochemical analysis of Plexin C1 in HCC and normal liver tissues. IHC is performed on HCC TMA. Representative images from nontumoral adjacent (a, c) and HCC tissues (b, d) are shown. The magnifications of the images are 50X for a and b and 200X for c and d. (e) H-scores of HCC and nontumoral adjacent tissues are compared with paired Student's t-test (p<0.05) and results are shown with box and whisker plots.

Finally, we turned to the analysis of Plexin C1 protein expression in HCC tissues. We performed immunohistochemical staining of TMA slides containing 90-paired spots of tumoral and nontumoral tissues of HCC patients. Hepatic cirrhosis and HCC coexisted in 16 tissues, but data on the etiological background of nontumoral liver and other HCC tissues was not available. The clinicopathological characteristics of patients are given in Supplement Table 1. PE4 antibody selectively stained the membrane of hepatocytes and HCC tumor cells (Figure 4(a)), and tumor tissues had significantly higher Plexin C1 protein levels (p=0.0118) than adjacent nontumoral areas (Figure 4(b)). Correlation analyses of Plexin C1 expression with clinicopathological characteristics of the samples did not show any correlation between the Plexin C1 levels of tissues and age, sex, stage and survival status of patients (Table 1). However, a significant correlation was found between Plexin C1 reactivity and the grade of HCC tumors (p<0.05). Grade I, Grade I-II and Grade II were considered as well-differentiated, and Grade II-III and Grade III were considered as poorly differentiated HCC tumors. Strong Plexin C1 staining was found in 66.15% and 55% of HCC cases with well-differentiated and poorly differentiated tumors, respectively. In contrast, weakly stained cases were higher in poorly differentiated compared to well-differentiated HCCs (36% vs. 13.85%).

## 4. Discussion

Hepatocellular carcinoma is the most common type of liver cancers with a very poor prognosis [19]. Currently, radiology, serum alpha-fetoprotein (AFP) levels and liver biopsy in advanced cases are used in clinical practice for the diagnosis of HCC [20–22]. Isoforms of AFP such as AFP-L3 and other markers including fucosylated Golgi Protein 73 (FC-GP73), *α*-l-fucosidase (AFU) and squamous cell carcinoma antigen (SCCA) have been evaluated in combination or as a single indicator and showed a sensitivity superior to AFP [23]. However, new serum and histologic markers are required to increase the sensitivity and specificity of HCC diagnosis. Plexin C1 was first discovered in the nervous system as an axon guidance receptor protein and was associated with neuronal cell adhesion [24, 25]. Engagement of Plexin C1 with its Sema7A ligand inhibited the spreading and dentricity of melanocytes [10]. In relation with this finding, an immunohistochemistry study in melanoma patients showed decreased levels of Plexin C1 protein in metastatic cases but its moderate to strong expression in non-metastatic melanoma and nevus tissues [9]. Recently, the interaction of Plexin C1 with its ligand Sema 7A was shown to inhibit pulmonary melanoma metastasis [26]. In addition, both Plexin C1 and its positive regulator GAS5, a long non-coding RNA, were downregulated in glioma tissues and cell lines [27]. Mechanistically, exogenous GAS5 inhibited miR222, a repressor of* PLXNC1*, and upregulated Plexin C1, which in turn inactivated cell motility protein cofilin. Therefore, compelling evidence was provided about the metastasis and tumor suppressor roles of Plexin C1 in human tumors. However, the expression profile of Plexin C1 in HCC and its role in hepatocarcinogenesis have not been elucidated so far.

After our initial observations through gene expression databases, namely, Oncomine and EMBL-EBI Expression Atlas, that* PLXNC1* is upregulated in HCC, we generated a monoclonal antibody, clone PE4, and demonstrated its specificity for Plexin C1 in* PLXNC1* knockdown and control cell clones by Western blotting and immunofluorescence. These results suggested a reactivity of PE4 with both denatured and conformational forms of the protein. Exploration of mRNA and protein expression across HCC cell lines revealed that Plexin C1 clustered well-differentiated HCC cells with the epithelial phenotype. In contrast, HCC cells well known for their invasive behavior displayed barely detectable Plexin C1 levels [28]. These results were in line with the aforementioned reports indicating that Plexin C1 is a suppressor of cell motility and invasiveness, as well as with our* in silico* analyses showing increased expression of Plexin C1 in well-differentiated compared to poorly differentiated HCC cell lines. Plexin C1 expression in HCC and control liver tissues was assessed by immunohistochemistry studies using HCC tissue microarrays. Variable reactivity of PE4 with membrane Plexin C1 was observed in HCC and control tissues compared to nonreactive isotype control antibody staining. A statistically significant overexpression of Plexin C1 was found in HCC specimens compared to nontumoral adjacent liver tissues (p<0.05). In correlation analyses, we could not detect any association between Plexin C1 intensity and clinicopathological parameters such as age, sex, stage, cirrhosis background, and survival of patients. However, a significant correlation of Plexin C1 staining with grade of HCC tumors was found. As observed in the analysis of Plexin C1 expression in HCC cell lines, weakly stained tumors were higher in poorly differentiated HCCs (36%) compared to well-differentiated cases (13.85%). Despite the explicit difference of Plexin C1 expression between epithelial-like and mesenchymal-like HCC cell lines, both tumoral and nontumoral tissues of HCC cases displayed Plexin C1 reactivity, with a significant superiority of tumors to nonassociated tissues. However, the lower expression of Plexin C1 in high-grade tumors was in accordance with our* in vitro* observation that Plexin C1 segregates well-differentiated HCC cells. One can also speculate that HCC tumors do not lose completely their epithelial characteristics and do not downregulate Plexin C1 extensively. Importantly, our results provide first evidence for the overexpression of Plexin C1 in tumors of epithelial origin. Specific membrane staining of Plexin C1 with PE4 antibody in paraffin-embedded HCC tissues further validated the usability of this monoclonal antibody for the study of Plexin C1 expression in other malignancies. Given the possibility of proteolytic ectodomain shedding of this receptor protein, screening of sera and tissues in a larger cohort of HCC patients may establish a diagnostic and/or prognostic value for Plexin C1.

## 5. Conclusions

In summary, we generated a monoclonal antibody specific for Plexin C1 capable to detect membrane-localized protein in paraffin-embedded tumor tissues. Our investigation of HCC cell lines and tissues revealed that Plexin C1 segregates HCC tumors of epithelioid phenotype and is overexpressed in HCC tissues.

## Figures and Tables

**Table 1:** Correlation of Plexin C1 staining with clinicopathological characteristics of the patients.

		Plexin C1 Staining			
	*N*	Negative	Weak	Strong	*P*
**Age**					
≤60	65	10 (15.38%)	12 (18.46%)	43 (66.15%)	
>60	25	5 (20%)	6 (24%)	14 (56%)	0.67
**Gender**					
F	16	3 (18.75%)	4 (25%)	9 (56.25%)	
M	74	12 (16.22%)	14 (18.92%)	48 (64.86%)	0.799
**Stage**					
1&2	52	11 (21.15%)	8 (15.38%)	33 (63.46%)	
3	31	4 (12.9%)	8 (25.81%)	19 (61.29%)	0.398
**Grade**					
Well-Differentiated	65	13 (20%)	9 (13.85%)	43 (66.15%)	
Poorly-Differentiated	25	2 (8%)	9 (36%)	14 (56%)	0.043
**Disease Status**					
HCC with cirrhosis	16	5 (31.25%)	2 (12.5%)	9 (56.25%)	
HCC	74	10 (13.51%)	16 (21.62%)	48 (64.86%)	0.204

N: number of cases. Correlation analyses were carried out with chi-square test. Plexin C1 staining was classified according to H-scores as follows: 0 = negative; 1-6 = weak; 7-12 = strong.

## Data Availability

All data represented in this study are contained in the manuscript and supplementary information.

## Supplementary Materials

Supplementary Materials Supplement Table 1: Clinicopathological characteristics of patients included in Tissue Microarray consist of age, sex, tumor grade, TNM staging, histopathological diagnosis, and survival data. NAT: nonassociated tissue
