# Supplementary material for: Plexin C1 Marks Liver Cancer Cells with Epithelial Phenotype and Is Overexpressed in Hepatocellular Carcinoma
Source: Can J Gastroenterol Hepatol. 2018 Sep 19;2018:4040787. doi: 10.1155/2018/4040787 (PMC6169229; doi:10.1155/2018/4040787)
Supplement: Supplementary Materials — Supplement Table 1: Clinicopathological characteristics of patients included in Tissue Microarray consist of age, sex, tumor grade, TNM staging, histopathological diagnosis, and survival data. NAT: nonassociated tissue [file 4040787.f1.docx]

| **Pos** | **No.** | **Sex** | **Age** | **Organ** | **Pathology diagnosis** | **Grade** | **Stage** | **TNM** | **Type** | **Surgery Date** | **Survival Status** | **Survival Months** |
| --- | --- | --- | --- | --- | --- | --- | --- | --- | --- | --- | --- | --- |
| A1 | 1 | F | 60 | Liver | HCC | II | 3 | T3N0M0 | Tumor | 7.1.2010 | survival | 44 |
| A2 | 2 | F | 60 | Liver | NAT |  |  |  | NAT |  |  |  |
| A3 | 3 | M | 61 | Liver | HCC | II | 2 | T2N0M0 | Tumor | 11.1.2010 | deceased | 22 |
| A4 | 4 | M | 61 | Liver | NAT |  |  |  | NAT |  |  |  |
| A5 | 5 | M | 56 | Liver | HCC, with hepatic cirrhosis | I | 2 | T2N0M0 |  | 12.1.2010 | survival | 44 |
| A6 | 6 | M | 56 | Liver | NAT |  |  |  | NAT |  |  |  |
| A7 | 7 | M | 48 | Liver | HCC | II | 3 | T3N0M0 | Tumor | 12.1.2010 | deceased | 26 |
| A8 | 8 | M | 48 | Liver | NAT |  |  |  | NAT |  |  |  |
| A9 | 9 | M | 39 | Liver | HCC | II | 2 | T2N0M0 | Tumor | 13.1.2010 | deceased | 31 |
| A10 | 10 | M | 39 | Liver | NAT |  |  |  | NAT |  |  |  |
| A11 | 11 | M | 51 | Liver | HCC | II |  |  | Tumor | 14.1.2010 | deceased | 31 |
| A12 | 12 | M | 51 | Liver | NAT |  |  |  | NAT |  |  |  |
| A13 | 13 | M | 48 | Liver | HCC | III | 3 | T4N0M0 | Tumor | 25.1.2010 | deceased | 5 |
| A14 | 14 | M | 48 | Liver | NAT |  |  |  | NAT |  |  |  |
| A15 | 15 | M | 50 | Liver | HCC | II | 3 | T4N0M0 | Tumor | 25.1.2010 | deceased | 12 |
| A16 | 16 | M | 50 | Liver | NAT |  |  |  | NAT |  |  |  |
| A17 | 17 | F | 54 | Liver | HCC | II | 2 | T2N0M0 | Tumor | 28.1.2010 | deceased | 36 |
| A18 | 18 | F | 54 | Liver | NAT |  |  |  | NAT |  |  |  |
| B1 | 19 | F | 57 | Liver | HCC | II | 2 | T2N0M0 | Tumor | 29.1.2010 | deceased | 18 |
| B2 | 20 | F | 57 | Liver | NAT |  |  |  | NAT |  |  |  |
| B3 | 21 | F | 59 | Liver | HCC | I-II | 1 | T1N0M0 | Tumor | 4.2.2010 | survival | 43 |
| B4 | 22 | F | 59 | Liver | NAT |  |  |  | NAT |  |  |  |
| B5 | 23 | F | 66 | Liver | HCC | III | 2 | T2N0M0 | Tumor | 5.2.2010 | deceased | 11 |
| B6 | 24 | F | 66 | Liver | NAT |  |  |  | NAT |  |  |  |
| B7 | 25 | F | 53 | Liver | HCC | II-III | 2 | T2N0M0 | Tumor | 8.2.2010 | survival | 43 |
| B8 | 26 | F | 53 | Liver | NAT |  |  |  | NAT |  |  |  |
| B9 | 27 | F | 40 | Liver | HCC | III | 3 | T3N0M0 | Tumor | 11.2.2010 | survival | 43 |
| B10 | 28 | F | 40 | Liver | NAT |  |  |  | NAT |  |  |  |
| B11 | 29 | M | 50 | Liver | HCC | II-III | 3 | T3N0M0 | Tumor | 12.2.2010 | deceased | 26 |
| B12 | 30 | M | 50 | Liver | NAT |  |  |  | NAT |  |  |  |
| B13 | 31 | M | 50 | Liver | HCC | II | 3 | T3N0M0 | Tumor | 12.2.2010 | deceased | 4 |
| B14 | 32 | M | 50 | Liver | NAT |  |  |  | NAT |  |  |  |
| B15 | 33 | M | 50 | Liver | HCC | II |  |  | Tumor | 2.3.2010 | deceased | 2 |
| B16 | 34 | M | 50 | Liver | NAT |  |  |  | NAT |  |  |  |
| B17 | 35 | M | 60 | Liver | HCC | II |  |  | Tumor | 3.3.2010 | deceased | 19 |
| B18 | 36 | M | 60 | Liver | NAT |  |  |  | NAT |  |  |  |
| C1 | 37 | F | 55 | Liver | HCC | II |  |  | Tumor | 26.3.2010 | deceased | 14 |
| C2 | 38 | F | 55 | Liver | NAT |  |  |  | NAT |  |  |  |
| C3 | 39 | M | 56 | Liver | HCC | II | 3 | T3N0M0 | Tumor | 2.4.2010 | deceased | 37 |
| C4 | 40 | M | 56 | Liver | NAT |  |  |  | NAT |  |  |  |
| C5 | 41 | F | 71 | Liver | HCC, with hepatic cirrhosis | II | 2 | T2N0M0 |  | 19.4.2010 | survival | 41 |
| C6 | 42 | F | 71 | Liver | NAT |  |  |  | NAT |  |  |  |
| C7 | 43 | M | 51 | Liver | HCC | II | 2 | T2N0M0 | Tumor | 23.4.2010 | deceased | 6 |
| C8 | 44 | M | 51 | Liver | NAT |  |  |  | NAT |  |  |  |
| C9 | 45 | M | 73 | Liver | HCC | I-II | 3 | T3N0M0 | Tumor | 6.5.2010 | deceased | 32 |
| C10 | 46 | M | 73 | Liver | NAT |  |  |  | NAT |  |  |  |
| C11 | 47 | M | 55 | Liver | HCC | I-II | 2 | T2N0M0 | Tumor | 7.5.2010 | survival | 40 |
| C12 | 48 | M | 55 | Liver | NAT |  |  |  | NAT |  |  |  |
| C13 | 49 | M | 46 | Liver | HCC | III | 2 | T2N0M0 | Tumor | 7.5.2010 | deceased | 19 |
| C14 | 50 | M | 46 | Liver | NAT |  |  |  | NAT |  |  |  |
| C15 | 51 | M | 39 | Liver | HCC | II-III | 3 | T3N0M0 | Tumor | 17.5.2010 | deceased | 36 |
| C16 | 52 | M | 39 | Liver | NAT |  |  |  | NAT |  |  |  |
| C17 | 53 | M | 55 | Liver | HCC | I-II | 2 | T2N0M0 | Tumor | 27.5.2010 | survival | 40 |
| C18 | 54 | M | 55 | Liver | NAT |  |  |  | NAT |  |  |  |
| D1 | 55 | M | 41 | Liver | HCC | II-III | 3 | T3N0M0 | Tumor | 1.6.2010 | deceased | 26 |
| D2 | 56 | M | 41 | Liver | NAT |  |  |  | NAT |  |  |  |
| D3 | 57 | M | 56 | Liver | HCC, with hepatic cirrhosis | II-III | 3 | T3N0M0 |  | 7.6.2010 | deceased | 0 |
| D4 | 58 | M | 56 | Liver | NAT |  |  |  | NAT |  |  |  |
| D5 | 59 | M | 56 | Liver | HCC, with hepatic cirrhosis | I-II | 1 | T1N0M0 |  | 8.6.2010 | survival | 39 |
| D6 | 60 | M | 56 | Liver | NAT |  |  |  | NAT |  |  |  |
| D7 | 61 | M | 62 | Liver | HCC | II-III | 2 | T2N0M0 | Tumor | 9.6.2010 | survival | 39 |
| D8 | 62 | M | 62 | Liver | NAT |  |  |  | NAT |  |  |  |
| D9 | 63 | M | 28 | Liver | HCC, with hepatic cirrhosis | II | 2 | T2N0M0 |  | 15.6.2010 | deceased | 7 |
| D10 | 64 | M | 28 | Liver | NAT |  |  |  | NAT |  |  |  |
| D11 | 65 | M | 46 | Liver | HCC | II-III |  |  | Tumor | 15.6.2010 | deceased | 10 |
| D12 | 66 | M | 46 | Liver | NAT |  |  |  | NAT |  |  |  |
| D13 | 67 | M | 48 | Liver | HCC, with hepatic cirrhosis | II | 3 | T3N0M0 |  | 25.6.2010 | deceased | 8 |
| D14 | 68 | M | 48 | Liver | NAT |  |  |  | NAT |  |  |  |
| D15 | 69 | M | 44 | Liver | HCC, with hepatic cirrhosis | II | 2 | T2N0M0 |  | 30.6.2010 | survival | 39 |
| D16 | 70 | M | 44 | Liver | NAT |  |  |  | NAT |  |  |  |
| D17 | 71 | F | 66 | Liver | HCC | II | 2 | T2N0M0 | Tumor | 30.6.2010 | survival | 39 |
| D18 | 72 | F | 66 | Liver | NAT |  |  |  | NAT |  |  |  |
| E1 | 73 | M | 53 | Liver | HCC, with hepatic cirrhosis | II | 2 | T2N0M0 |  | 13.7.2010 | survival | 38 |
| E2 | 74 | M | 53 | Liver | NAT |  |  |  | NAT |  |  |  |
| E3 | 75 | M | 47 | Liver | HCC, with hepatic cirrhosis | II | 3 | T3N0M0 |  | 19.7.2010 | deceased | 9 |
| E4 | 76 | M | 47 | Liver | NAT |  |  |  | NAT |  |  |  |
| E5 | 77 | M | 53 | Liver | HCC | II | 3 | T3N0M0 | Tumor | 20.7.2010 | survival | 38 |
| E6 | 78 | M | 53 | Liver | NAT |  |  |  | NAT |  |  |  |
| E7 | 79 | M | 54 | Liver | HCC | III | 3 | T3N0M0 | Tumor | 22.7.2010 | deceased | 10 |
| E8 | 80 | M | 54 | Liver | NAT |  |  |  | NAT |  |  |  |
| E9 | 81 | M | 63 | Liver | HCC | II-III | 2 | T2N0M0 | Tumor | 28.7.2010 | deceased | 12 |
| E10 | 82 | M | 63 | Liver | NAT |  |  |  | NAT |  |  |  |
| E11 | 83 | M | 44 | Liver | HCC, with hepatic cirrhosis | II | 2 | T2N0M0 |  | 5.8.2010 | survival | 37 |
| E12 | 84 | M | 44 | Liver | NAT |  |  |  | NAT |  |  |  |
| E13 | 85 | M | 72 | Liver | HCC, with hepatic cirrhosis | II | 1 | T1N0M0 |  | 6.8.2010 | survival | 37 |
| E14 | 86 | M | 72 | Liver | NAT |  |  |  | NAT |  |  |  |
| E15 | 87 | F | 60 | Liver | HCC, with hepatic cirrhosis | II | 2 | T2N0M0 |  | 20.8.2010 | deceased | 11 |
| E16 | 88 | F | 60 | Liver | NAT |  |  |  | NAT |  |  |  |
| E17 | 89 | F | 47 | Liver | HCC | II-III | 2 | T2N0M0 | Tumor | 25.8.2010 | survival | 37 |
| E18 | 90 | F | 47 | Liver | NAT |  |  |  | NAT |  |  |  |
| F1 | 91 | M | 45 | Liver | HCC | II | 2 | T2N0M0 | Tumor | 3.9.2010 | survival | 36 |
| F2 | 92 | M | 45 | Liver | NAT |  |  |  | NAT |  |  |  |
| F3 | 93 | M | 53 | Liver | HCC | II | 2 | T2N0M0 | Tumor | 26.9.2010 | survival | 36 |
| F4 | 94 | M | 53 | Liver | NAT |  |  |  | NAT |  |  |  |
| F5 | 95 | M | 53 | Liver | HCC | II | 3 | T3N0M0 | Tumor | 30.9.2010 | deceased | 20 |
| F6 | 96 | M | 53 | Liver | NAT |  |  |  | NAT |  |  |  |
| F7 | 97 | M | 43 | Liver | HCC | II | 2 | T2N0M0 | Tumor | 21.10.2010 | survival | 35 |
| F8 | 98 | M | 43 | Liver | NAT |  |  |  | NAT |  |  |  |
| F9 | 99 | M | 51 | Liver | HCC | II | 2 | T2N0M0 | Tumor | 10.11.2010 | survival | 34 |
| F10 | 100 | M | 51 | Liver | NAT |  |  |  | NAT |  |  |  |
| F11 | 101 | M | 58 | Liver | HCC | II-III | 3 | T3N0M0 | Tumor | 12.11.2010 | survival | 34 |
| F12 | 102 | M | 58 | Liver | NAT |  |  |  | NAT |  |  |  |
| F13 | 103 | F | 65 | Liver | HCC | II | 3 | T3N0M0 | Tumor | 30.11.2010 | survival | 34 |
| F14 | 104 | F | 65 | Liver | NAT |  |  |  | NAT |  |  |  |
| F15 | 105 | M | 48 | Liver | HCC | III | 2 | T2N0M0 | Tumor | 4.1.2011 | deceased | 25 |
| F16 | 106 | M | 48 | Liver | NAT |  |  |  | NAT |  |  |  |
| F17 | 107 | M | 61 | Liver | HCC | II | 3 | T3N0M0 | Tumor | 5.1.2011 | survival | 32 |
| F18 | 108 | M | 61 | Liver | NAT |  |  |  | NAT |  |  |  |
| G1 | 109 | M | 67 | Liver | HCC | III | 3 | T3N0M0 | Tumor | 10.1.2011 | deceased | 2 |
| G2 | 110 | M | 67 | Liver | NAT |  |  |  | NAT |  |  |  |
| G3 | 111 | M | 40 | Liver | HCC | II-III | 3 | T3N0M0 | Tumor | 11.1.2011 | deceased | 18 |
| G4 | 112 | M | 40 | Liver | NAT |  |  |  | NAT |  |  |  |
| G5 | 113 | M | 38 | Liver | HCC | II | 3 | T3N0M0 | Tumor | 13.1.2011 | deceased | 18 |
| G6 | 114 | M | 38 | Liver | NAT |  |  |  | NAT |  |  |  |
| G7 | 115 | F | 54 | Liver | HCC | II | 2 | T2N0M0 | Tumor | 25.1.2011 | survival | 32 |
| G8 | 116 | F | 54 | Liver | NAT |  |  |  | NAT |  |  |  |
| G9 | 117 | M | 62 | Liver | HCC | II | 2 | T2N0M0 | Tumor | 26.1.2011 | deceased | 0 |
| G10 | 118 | M | 62 | Liver | NAT |  |  |  | NAT |  |  |  |
| G11 | 119 | M | 56 | Liver | HCC | I-II | 2 | T2N0M0 | Tumor | 27.1.2011 | survival | 32 |
| G12 | 120 | M | 56 | Liver | NAT |  |  |  | NAT |  |  |  |
| G13 | 121 | M | 63 | Liver | HCC | II-III |  |  | Tumor | 14.2.2011 | deceased | 2 |
| G14 | 122 | M | 63 | Liver | NAT |  |  |  | NAT |  |  |  |
| G15 | 123 | M | 59 | Liver | HCC, with hepatic cirrhosis | I-II | 1 | T1N0M0 |  | 4.06.2011 | survival | 30 |
| G16 | 124 | M | 59 | Liver | NAT |  |  |  | NAT |  |  |  |
| G17 | 125 | M | 55 | Liver | HCC | II | 2 | T2N0M0 | Tumor | 21.3.2011 | deceased | 26 |
| G18 | 126 | M | 55 | Liver | NAT |  |  |  | NAT |  |  |  |
| H1 | 127 | M | 45 | Liver | HCC | II | 3 | T3N0M0 | Tumor | 24.3.2011 | deceased | 22 |
| H2 | 128 | M | 45 | Liver | NAT |  |  |  | NAT |  |  |  |
| H3 | 129 | M | 57 | Liver | HCC, with hepatic cirrhosis | I-II | 1 | T1N0M0 |  | 11.4.2011 | survival | 29 |
| H4 | 130 | M | 57 | Liver | NAT |  |  |  | NAT |  |  |  |
| H5 | 131 | M | 61 | Liver | HCC | II | 3 | T3N0M0 | Tumor | 19.4.2011 | survival | 29 |
| H6 | 132 | M | 61 | Liver | NAT |  |  |  | NAT |  |  |  |
| H7 | 133 | M | 61 | Liver | HCC | II | 2 | T2N0M0 | Tumor | 20.4.2011 | survival | 29 |
| H8 | 134 | M | 61 | Liver | NAT |  |  |  | NAT |  |  |  |
| H9 | 135 | M | 65 | Liver | HCC, with hepatic cirrhosis | II | 2 | T2N0M0 |  | 29.4.2011 | survival | 29 |
| H10 | 136 | M | 65 | Liver | NAT |  |  |  | NAT |  |  |  |
| H11 | 137 | F | 76 | Liver | HCC | II-III | 3 | T3N0M0 | Tumor | 17.5.2011 | survival | 28 |
| H12 | 138 | F | 76 | Liver | NAT |  |  |  | NAT |  |  |  |
| H13 | 139 | M | 47 | Liver | HCC | I | 2 | T2N0M0 | Tumor | 23.5.2011 | survival | 28 |
| H14 | 140 | M | 47 | Liver | NAT |  |  |  | NAT |  |  |  |
| H15 | 141 | M | 73 | Liver | HCC | I-II | 3 | T3N0M0 | Tumor | 23.5.2011 | deceased | 1 |
| H16 | 142 | M | 73 | Liver | NAT |  |  |  | NAT |  |  |  |
| H17 | 143 | M | 51 | Liver | HCC | III |  |  | Tumor | 26.5.2011 | deceased | 6 |
| H18 | 144 | M | 51 | Liver | NAT |  |  |  | NAT |  |  |  |
| I1 | 145 | M | 55 | Liver | HCC, with hepatic cirrhosis | II | 2 | T2N0M0 |  | 2.6.2011 | survival | 27 |
| I2 | 146 | M | 55 | Liver | NAT |  |  |  | NAT |  |  |  |
| I3 | 147 | M | 49 | Liver | HCC | I-II | 2 | T2N0M0 | Tumor | 3.6.2011 | survival | 27 |
| I4 | 148 | M | 49 | Liver | NAT |  |  |  | NAT |  |  |  |
| I5 | 149 | M | 53 | Liver | HCC | I-II | 3 | T3N0M0 | Tumor | 16.6.2011 | survival | 27 |
| I6 | 150 | M | 53 | Liver | NAT |  |  |  | NAT |  |  |  |
| I7 | 151 | M | 48 | Liver | HCC | II-III | 2 | T2N0M0 | Tumor | 27.6.2011 | survival | 27 |
| I8 | 152 | M | 48 | Liver | NAT |  |  |  | NAT |  |  |  |
| I9 | 153 | M | 68 | Liver | HCC | III | 3 | T3N0M0 | Tumor | 28.6.2011 | deceased | 14 |
| I10 | 154 | M | 68 | Liver | NAT |  |  |  | NAT |  |  |  |
| I11 | 155 | M | 66 | Liver | HCC | III | 2 | T2N0M0 | Tumor | 6.7.2011 | survival | 26 |
| I12 | 156 | M | 66 | Liver | NAT |  |  |  | NAT |  |  |  |
| I13 | 157 | M | 64 | Liver | HCC | II | 2 | T2N0M0 | Tumor | 15.7.2011 | survival | 26 |
| I14 | 158 | M | 64 | Liver | NAT |  |  |  | NAT |  |  |  |
| I15 | 159 | M | 54 | Liver | HCC | II | 2 | T2N0M0 | Tumor | 19.7.2011 | deceased | 24 |
| I16 | 160 | M | 54 | Liver | NAT |  |  |  | NAT |  |  |  |
| I17 | 161 | M | 66 | Liver | HCC | II | 2 | T2N0M0 | Tumor | 25.7.2011 | deceased | 19 |
| I18 | 162 | M | 66 | Liver | NAT |  |  |  | NAT |  |  |  |
| J1 | 163 | M | 63 | Liver | HCC | I | 1 | T1N0M0 | Tumor | 25.7.2011 | survival | 26 |
| J2 | 164 | M | 63 | Liver | NAT |  |  |  | NAT |  |  |  |
| J3 | 165 | M | 39 | Liver | HCC | I-II | 1 | T1N0M0 | Tumor | 8.8.2011 | survival | 25 |
| J4 | 166 | M | 39 | Liver | NAT |  |  |  | NAT |  |  |  |
| J5 | 167 | M | 66 | Liver | HCC | I-II | 2 | T2N0M0 | Tumor | 10.8.2011 | deceased | 23 |
| J6 | 168 | M | 66 | Liver | NAT |  |  |  | NAT |  |  |  |
| J7 | 169 | M | 49 | Liver | HCC | II-III | 2 | T2N0M0 | Tumor | 12.8.2011 | deceased | 9 |
| J8 | 170 | M | 49 | Liver | NAT |  |  |  | NAT |  |  |  |
| J9 | 171 | M | 37 | Liver | HCC | II | 3 | T3N0M0 | Tumor | 25.8.2011 | survival | 25 |
| J10 | 172 | M | 37 | Liver | NAT |  |  |  | NAT |  |  |  |
| J11 | 173 | M | 65 | Liver | HCC | I-II | 2 | T2N0M0 | Tumor | 25.8.2011 | survival | 25 |
| J12 | 174 | M | 65 | Liver | NAT |  |  |  | NAT |  |  |  |
| J13 | 175 | M | 59 | Liver | HCC | II | 2 | T2N0M0 | Tumor | 26.8.2011 | survival | 25 |
| J14 | 176 | M | 59 | Liver | NAT |  |  |  | NAT |  |  |  |
| J15 | 177 | M | 55 | Liver | HCC | II | 2 | T2N0M0 | Tumor | 30.8.2011 | deceased | 24 |
| J16 | 178 | M | 55 | Liver | NAT |  |  |  | NAT |  |  |  |
| J17 | 179 | F | 43 | Liver | HCC | II | 3 | T3N0M0 | Tumor | 2.9.2011 | survival | 24 |
| J18 | 180 | F | 43 | Liver | NAT |  |  |  | NAT |  |  |  |
